# A visual tutorial on the synthesis of gold nanoparticles

**DOI:** 10.2349/biij.6.1.e9

**Published:** 2010-01-01

**Authors:** A Low, V Bansal

**Affiliations:** Medical Radiationse, School of Medical Sciences, RMIT University, Victoria, Australia

**Keywords:** gold nanoparticles, video tutorial, synthesis

## Abstract

Many papers have been written on the synthesis of gold nanoparticles but very few included pictures of the process, and none of them used video to show the whole process of synthesis. This paper records the process of synthesis of gold nanoparticles using video clips. Every process from cleaning of glassware, an important step in the synthesis of metallic nanoparticles, to the dialysis process is shown. It also includes the preparation of aqua regia and the actual synthesis of gold nanoparticles. In some papers, the dialysis process was omitted, but in this paper, it is included to complete the whole process as it is being used for purification.

## INTRODUCTION

There were many reports in journal articles on the synthesis of gold nanoparticles (AuNPs) [1-26]. However, very few images were shown and none of them showed any video clips detailing the whole process of making AuNPs.

There were variations in the process of synthesising AuNPs. Chloroauric acid (HAuCl_4_) is typically used as the reactant containing gold atoms [1-26], and most of them reported to use trisodium citrate [1-4, 8, 17, 19, 23, 25] or sodium borohydride (NaBH_4_) [5, 7, 9-16], as the reducing agent. In this experiment, NaBH_4_ was used as the reducing agent.

This paper attempts to illustrate the process of synthesis of AuNPs with video clips. As a picture speaks a thousand words, so a video speaks ten-thousand words.

## METHODS AND MATERIALS

### The process

500 mL Milli-Q H_2_O + 1 mL of 10-1 M HAuCl_4_ + 0.05 g NaBH_4_ ==> 0.2 mM AuNPs

### The laboratory

The laboratory consists of glassware, fume cupboard, deionised water, Milli-Q water, balance, hot plate with magnetic stirrer, pipette, etc (video 1).

**Video 1:** Laboratory facilities.

### The preparation of aqua regia solution

**Figure 1:**
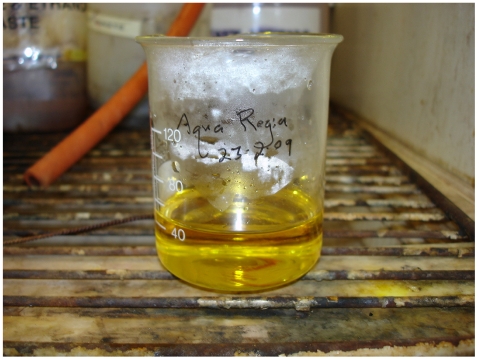
Aqua regia solution.

Aqua regia was prepared by mixing 3 parts hydrochloric acid (HCl) to 1 part nitric acid (HNO_3_) by volume [1-6, 24, 25] in a beaker (figure 1, video 2). Both items were obtained from Merck Pty Limited. Aqua regia should be prepared just before its use as it will lose its effectiveness quickly. Aqua regia is corrosive and highly oxidising. It should be prepared in a well-ventilated fume cupboard with protective clothing, goggles and gloves. It is used for cleaning glassware as it can dissolve any residual metallic particles, which may interfere with the synthesis.

**Video 2:** Aqua regia.

### The cleaning process

The cleaning of glassware and experiment utensils is a laborious process before AuNPs can be synthesised. Detergent and aqua regia were used to clean all the glassware, rinsing was done with deionised water and final washing in Milli-Q water (video 3, video 4).

**Video 3:** Wash with detergent.

**Video 4:** Wash with aqua regia and deionised water.

### The synthesis process

HAuCl_4_ and NaBH_4_ were purchased from Aldrich (America). Milli-Q water was used for the preparation of the solution for this experiment. Milli-Q water is deionised water, which has been further purified by Milli-Q purification system [6-8]. However, quite a few reported just using deionised water instead of Milli-Q water [9-11].

Then, 0.05 g of NaBH_4_ is added to 10 mL of Milli-Q water. The centrifuge tube was weighed first followed by NaBH_4._ In the process of getting the correct amount of NaBH_4_, 0.06 g of it was weighed instead. In order to get the same concentration, 12 mL of water was added to obtain the same concentration level. The solution in the tube was shaken to ensure that all the NaBH_4_ was dissolved (video 5).

**Video 5:** Get NaBH_4_ concentration.

In order to obtain 500 mL of 0.2 mM amount of naked gold nanoparticles, 500 mL of Milli-Q water is poured into a flask. Using a pipette, 1 mL amount of 0.1 M HAuCl_4_ aqueous solution, yellow in colour, was transferred to the flask. It was then shaken to mix the solution well (video 6).

**Video 6:** Prepare HAuCl_4_ solution.

**Figure 2:**
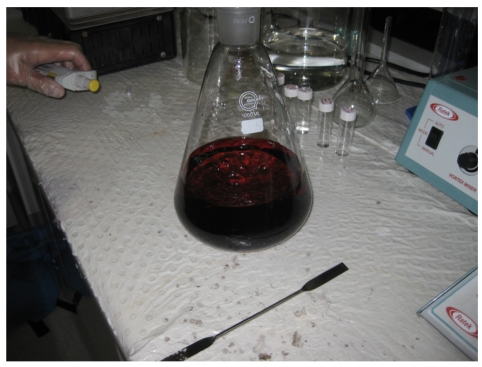
AuNPs.

NaBH_4_ was added as a reductant [7, 9-16] to obtain naked gold nanoparticles. Using a pipette, 10 mL of NaBH_4_ solution was transferred dropwise to the flask. It was added slowly initially to prevent aggregation and, subsequently, could be added more quickly. It was shaken well in the flask for each aliquot of reducing agent added. The solution in the flask should change from yellowish to ruby red in colour. The ruby red colour indicates the formation of gold nanoparticles [17] (figure 2, video 7).

**Video 7:** To obtain AuNPs.

### The dialysis process

**Figure 3:**
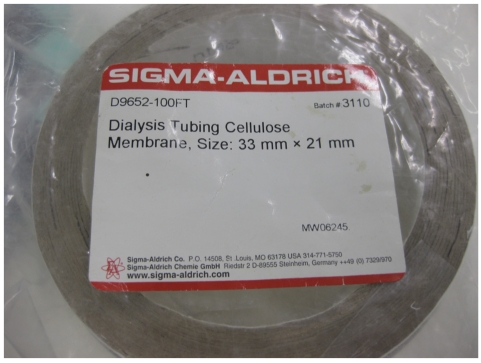
Dialysis tubing cellulose membrane.

The dialysis is the last process. Dialysis tubing cellulose membrane from Sigma Aldrich was used in this dialysis process. In short, the dialysis tubing cellulose membrane is called the dialysis bag (figure 3).

The outside and inside of the dialysis bag was washed with 20 mL of deionised water and then put in a beaker with a magnetic stirrer to boil for about 5 minutes. The water was poured out and the step repeated with another 20 mL of deionised water (video 8).

**Video 8:** Dialysis bag wash and boil.

The boiled water was poured away and the dialysis bag was washed with Milli-Q water (video 9).

**Video 9:** Dialysis bag wash with Milli-Q water.

The dialysis bag was pressed between the fingers to remove as much water in the tubing as possible. One end was folded and clipped with a peg. A funnel, which was cleaned with aqua regia solution, was used to pour AuNPs into it. The bag was tested for leakage before further AuNPs suspension was poured into it. Once all the liquid was transferred, the other end of the tubing was also folded and clipped. The dialysis bag was then placed in a large beaker (video 10).

**Video 10:** Fill dialysis bag with AuNPs.

The beaker with the dialysis membrane was filled with Milli-Q water. The more water is filled, the quicker it would be for the purification of AuNPs (video 11).

**Video 11:** Submerged dialysis bag with Milli-Q.

It was boiled for 6-hourly and the water was changed three times. It will yield 0.2 mM concentration of gold nanoparticles (video 12).

**Video 12:** Boil for 6-hourly.

## DISCUSSION

There were variations in the types of water being used in the synthesis of AuNPs. Some used deionised water throughout the experiments [8-11], others used doubly distilled water [4, 20, 22], nanopure water [1-3], ultrapure water [25], and Milli-Q water [5-7, 15, 19, 21, 23]. In this experiment, deionised water and Milli-Q water were used.

Dialysis is to purify the resultant solution [10] and to remove the extra free small molecules [4, 9]. However, many synthesis AuNPs without going through the dialysis process [1-3, 5-8, 11-25]. Experiments of synthesising AuNPs were conducted with and without the dialysis process, and both AuNPs solutions looked visibly the same after 3 months of synthesising.

The size of the AuNPs could be analysed by transmission electron microscope [1, 2, 6-9, 15, 17, 21, 22]. However, different sizes of AuNPs were prepared by altering the ratio of HAuCl_4_ and the reducing agent [26].

## CONCLUSION

This paper visually describes each stages of AuNPs synthesis from the preparation of aqua regia solution to the dialysis of the final suspension. The purpose of the last process, i.e., the dialysis, was to demonstrate the complete process of synthesis even though some of the authors do not find this process necessary.
